# Editorial: Insights in neuromorphic engineering: 2021

**DOI:** 10.3389/fnins.2023.1162831

**Published:** 2023-03-13

**Authors:** André van Schaik, Bernabé Linares-Barranco

**Affiliations:** ^1^The MARCS Institute, International Centre for Neuromorphic Systems, Western Sydney University, Penrith, NSW, Australia; ^2^Instituto de Microelectrónica de Sevilla (IMSE-CNM), CSIC and Universidad de Sevilla, Sevilla, Spain

**Keywords:** neuromorphic, insights 2021, SNN, sensing, benchmarks

In 2021 Frontiers organized a series of Research Topics to highlight the latest advancements in order to be at the forefront of science in different fields of research. As the Specialty Chief Editors of the Neuromorphic Engineering section, we organized on to focus on new insights, novel developments, current challenges, latest discoveries, and recent advances in the field of Neuromorphic Engineering. We solicited brief, forward-looking contributions from the editorial board members that describe the state of the art, outlining, recent developments and major accomplishments that have been achieved and that need to occur to move the field forward. Authors were encouraged to identify the greatest challenges in the sub-disciplines, and how to address those challenges. We have collected six articles in this Research Topic.

Davidson and Furber, in “*Comparison of artificial and spiking neural networks on digital hardware*”, establish a simple method of assessing the relative advantages of rate-based spike encoding over a conventional ANN model. Assuming identical underlying silicon technology they show that most rate-coded spiking network implementations will not be more energy or resource efficient than the original ANN, concluding that more imaginative uses of spikes are required to displace conventional ANNs as the dominant computing framework for neural computation.

Parlevliet et al., “*Autonomous flying with neuromorphic sensing*” argue for a bio-inspired approach to solve autonomous flying challenges, outline the frontier of sensing, data processing, and flight control within a neuromorphic paradigm, and chart directions of research needed to achieve operational capabilities comparable to those we observe in nature. They provide recommendations for sensor hardware and processing algorithm development to enable energy efficient and computationally effective flight control.

In Milde et al., “*Neuromorphic engineering needs closed-loop benchmarks*”, researchers from the International Centre for Neuromorphic Systems make the case for a renewed focus on closed-loop benchmarks involving real-world tasks, because such benchmarks will be crucial in developing and progressing neuromorphic Intelligence. They argue that the shift toward dynamic real-world benchmarking tasks should usher in richer, more resilient, and robust artificially intelligent systems in the future.

Yang et al., “*SAM: A unified self-adaptive multicompartmental spiking neuron model for learning with working memory*” present a self-adaptive multicompartment spiking neuron model that integrates four major biological principles: sparse coding, dendritic non-linearity, intrinsic self-adaptive dynamics, and spike-driven learning. Their experimental results highlight the energy efficiency and robustness of SAM in a wide range of challenging tasks, showing that SAM neurons may be used to develop efficient adaptive neuromorphic computing systems.

“*Automotive radar processing with spiking neural networks: Concepts and challenges*” by Vogginger et al., was the most viewed Frontiers in Neuroscience article in 2022. The article performs a step-by-step analysis of automotive radar processing and demonstrates how spiking neural networks could replace or complement conventional radar processing. They describe two SNN implementations, one for radar target detection, and another for radar target classification, and demonstrate a significantly reduced computational cost, promising more energy efficient realizations of radar processing for autonomous vehicles.

Finally, Bonilla et al., “*Analyzing time-to-first-spike coding schemes: A theoretical approach*” introduces a new unifying mathematical framework that allows the comparison of various time-to-first-spike (TTFS) information coding schemes. They use this framework to compare three TTFS schemes, and conclude that the Ranked N-of-M (R-NoM) scheme, proposed as a novel scheme in this article, performs best. The R-NoM scheme is easy to implement in neuromorphic hardware.

Our goal with this Research Topic was to shed light on the progress made in the past decade in Neuromorphic Engineering and on its challenges to provide a thorough overview of the status of the art of the field. We can see that, although research is still required to improve effectiveness and performance, neuromorphic systems are continuously becoming more efficient and some specific real-world applications are beginning to be demonstrated. We hope it will inspire, inform and provide direction and guidance to researchers in the field.

## Author contributions

BL-B and AS co-wrote this editorial. All authors contributed to the article and approved the submitted version.

